# The age factor influencing long-term physical functionality in stroke patients undergoing intra-arterial thrombectomy treatment

**DOI:** 10.1097/MD.0000000000030712

**Published:** 2022-09-23

**Authors:** Chi-Ling Kao, Chih-Ming Lin, Shu-Wei Chang, Chi-Kuang Liu, Yang-Hao Ou, Henry Horng-Shing Lu

**Affiliations:** a Institute of Statistics, National Yang Ming Chiao Tung University, Hsinchu City, Taiwan; b Department of Neurology, Changhua Christian Hospital, Changhua City, Taiwan; c Department of Medicinal Botanicals and Foods on Health Applications, Dayeh University, Changhua County, Taiwan; d Department of Social Work and Child Welfare, Providence University, Taichung City, Taiwan; e Graduate Institute of Statistics and Information Science, National Changhua University of Education, Changhua City, Taiwan; f Department of Post-Baccalaureate Medicine, College of Medicine, National Chung Hsing University, Taichung, Taiwan; g Department of Medical Imaging, Changhua Christian Hospital, Changhua City, Taiwan.

**Keywords:** acute ischemic stroke, age threshold, Barthel index, functional outcome, intra-arterial thrombectomy, intravenous thrombolysis, mRS score, NIHSS score

## Abstract

The treatment of acute ischemic stroke is heavily time-dependent, and even though, with the most efficient treatment, the long-term functional outcome is still highly variable. In this current study, the authors selected acute ischemic stroke patients who were qualified for intravenous thrombolysis with recombinant tissue plasminogen activator and followed by intra-arterial thrombectomy. With primary outcome defined by the functional level in a 1-year follow-up, we hypothesize that patients with older age are at a disadvantage in post-stroke recovery. However, an age-threshold should be determined to help clinicians in selection of patients to undergo such therapy. This is a retrospective chart review study that include 92 stroke patients in Changhua Christian hospital with a total of 68 evaluation indexes recorded. The current study utilized the forward stepwise regression model whose Adj-*R*^2^ and *P* value in search of important variables for outcome prediction. The chngpt package in R indicated the threshold point of the age factor directing the better future functionality of the stroke patients. Datasets revealed the threshold of the age set at 79 the most appropriate. Admission Barthel Index, Age, ipsilateral internal carotid artery resistance index (ICA RI), ipsilateral vertebral artery (VA) PI, contralateral middle cerebral artery (MCA) stenosis, contralateral external carotid artery (ECA) RI, and in-hospital pneumonia are the significant predicting variables. The higher the age, in-hospital pneumonia, contralateral MCA stenosis, ipsilateral ICA RI and ipsilateral VA PI, the less likely patient to recover from functional deficits as the result of acute ischemic stroke; the higher the value of contralateral ECA RI and admission Barthel Index, the better chance to full functional recovery at 1-year follow up. Parameters of pre-intervention datasets could provide important information to aid first-line clinicians in decision making. Especially, in patients whose age is above 79 receives diminish return in the benefit to undergo such intervention and should be considered seriously by both the patients and the physicians.

## 1. Introduction

The treatment of acute ischemic stroke is heavily time-dependent, and even though, with the most efficient treatment, the long-term functional outcome is still highly variable. In general, there are 3 clusters of parameters that influence the treatment outcome; these are the pre-intervention baseline characteristics, such as age, sex, body weight, co-morbidities, nature of the stroke, etc; secondly, interventional methods; and lastly, post-interventional care, including method of secondary prevention, rehabilitation program and much more. Though the advent of current stroke therapy guideline abide the bridging therapy as gold standard treatment, there is still a lack of knowledge as to the suitability and long term functional assessment in terms of threshold of age.^[1]^

In this current study, the authors selected acute ischemic stroke patients who were qualified for intravenous thrombolysis with recombinant tissue plasminogen activator and followed by intra-arterial thrombectomy, following the 2018 American Heart and Stroke Association’s guidelines.^[1,2]^ Additionally, all of the patients who underwent intra-arterial thrombectomy in Changhua Christian Hospital also received carotid doppler examination as part of the protocol. This offers additional data for evaluation in this study.

The primary goal of this study was to investigate whether pre-thrombectomy treatment parameters and baseline characteristics can offer valuable information in predicting patients’ long-term functional outcomes in acute ischemic stroke patients who underwent intravenous thrombolysis followed by intra-arterial thrombectomy. Secondly, we attempt to finalize the age threshold as to stroke bridging therapy, which allowing clinicians to better select the appropriate candidate embracing favorable outcomes.

## 2. Material and Methods

### 2.1. Patient selection and enrolled criteria

In this current study, all patients were selected from Changhua Christian Hospital with a retrospective chart review conducted by the Department of Neurology. Participants were selected based on the confirmed diagnosis of first acute ischemic stroke and underwent intravenous thrombolysis therapy with recombinant tissue plasminogen activator (rtPA), followed by intra-arterial thrombectomy. Patients were selected based on the current indication for intravenous thrombolysis of presenting within 4.5 hours of the onset of symptoms and did not have any contraindications to receive rtPA. CT angiography perfusion scan was followed in these patients to screen for candidates to receive intra-arterial thrombectomy.

**Figure 1.**
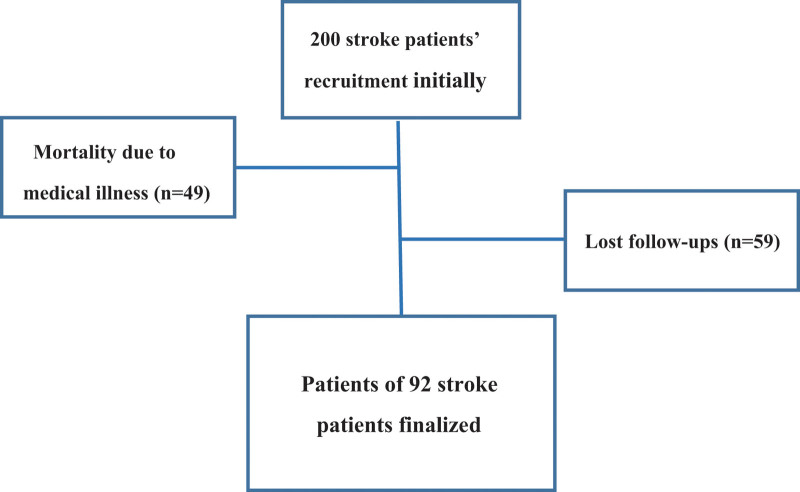
Research flow chart

Additional inclusion criteria are neuroimaging confirmed anterior circulation obstruction, above 18 of age, non-recurrent stroke was documented of and were followed for at least 1 year. Patients with intracerebral hemorrhage, aneurysm rupture, cerebral arteriovenous malformation, and recurrent stroke were excluded. Initially, there are 200 patients fitted in to the inclusion and exclusion criteria. With 50 patients lost follow-ups, 49 patients mortality due to the internal medicine diseases, and, 9 patients’ dropped out of the study due to recurrent stroke episode, that lead to the total 92 patients was selected fulfilling the study protocol the year of between 2015 and 2017 (please refer to Fig. 1).

Pre-intervention data include baseline patient demographics such as age, body mass index, systolic and diastolic blood pressure, total cholesterol, HbA1c, and other comorbidities were documented. Additionally, in-hospital complication such as pneumonia was also recorded.

### 2.2. Study ethical issue

The study procedures were approved by the Institutional Review Board of the Changhua Christian Hospital in Changhua, Taiwan (CCH IRB number: 180409). Informed consent was obtained in the usual way as the procedures were the usual standard of care. As the study is retrospective in nature, the informed consent was waived and approved by the committee. The stored patients’ information was well stored at the Basement 2 of Department of neuroimaging. Locker room, which was in charge by Dr Chi-Kuang Liu.

### 2.3. Intravenous thrombolytic therapy

All patients were evaluated for adequacy of thrombolytic therapy (rtPA) at the ED by the in-charge physician (The window of time for the rtPA was set at ≦ 4.5 hours from symptom onset) A thorough evaluation of every single patient was carried out before the administration of r-tPA, including neuroimaging, and the patient’s stroke severity was recorded using the NIHSS score. Shortly after the r-tPA, the patient’s baseline specific aspects were documented, together with the demographic information, NIHSS score, Barthel index, mRS, and blood biochemistry.

### 2.4. Intra-arterial thrombectomy

The indications for IAT were findings of major artery stenosis (visualized and confirmed by a neuroradiologist) with a suitable access location in order for the procedure to be carried out, that is, terminal intracranial ICA, and the first or second branch of the MCA. The IAT procedure was performed with informed consent from the patient or family. One 8F right femoral sheath was inserted and 1 6F Neuro Max long sheath was advanced up to the stenotic site, then 1 3Max Penumbra aspiration reperfusion catheter was inserted coaxially via the guiding catheter and navigated to the proximal part of the occluded MCA to remove the thrombus.

A post-procedural immediate outcome was evaluated by a neuroradiologist (Dr Chi Kuang Liu) using the modified thrombolysis in cerebral infarction (mTICI) score. The TICI score was defined as the following: 0 = no perfusion; 1 = penetration, but no distal branch filling; 2a = perfusion with incomplete (<50%) distal branch filling; 2b = perfusion with incomplete (>50%) distal branch filling; and 3 = full reperfusion with filling of all distal branches. A satisfactory result was defined as a TICI score of 2b or 3.^[3]^

### 2.5. Cervical carotid duplex exam

All procedures were conducted by a stoke neurologist team in a specialized angiography clinic at the Changhua Christian Hospital, Taiwan. Cervical carotid artery ultrasound examination (Philips iE33 7-Mhz linear transducer) was performed upon the patient once upon arriving at the emergency room. Cross-sectional B-mode scanning was performed to check for intraluminal plaque material and the longitudinal screening method was adopted to confirm the presence of plaque. Two physicians assessed and classified plaques into subtypes 1, 2, 3, or 4 according to the International Classification System.^[4]^ Whenever there was a disagreement between the physicians, a third physician would make the final assessment. The intima-media thickness of the mid-portion of the common carotid artery was measured on the ipsilateral side of the index stroke event. The parameters of peak systolic velocity (PSV), end diastolic velocity (EDV), resistance index (RI) (calculated as [PSV-EDV]/PSV) and pulsatility index (PI) (calculated as [PSV-EDV]/mean of the velocity) of the common carotid artery, ICA, ECA, VA, and OA were measured bilaterally. The reversal of blood flow in the OA was also measured. Forward flow was defined as blood flow detected out of the stenotic ipsilateral carotid artery, whereas reverse flow was defined as blood flow into the carotid artery.^[5,6]^ The machine Philips iE33 7 automatically calculated the plaque index on both the ipsilateral and contralateral sides of the cerebral lesion.^[9]^ The approximate time of assessing the extracranial artery blood flows by carotid duplex was 30 minutes.

### 2.6. Cerebral computed tomography (CT)/angiography (CTa), and/CT perfusion (P)

CTA examinations were performed using a second-generation dual-source CT scanner (SOMATOM Definition Flash, Siemens Healthcare, Forchheim, Germany). Perfusion data sets were post-processed on a Siemens Multimodality Workplace Workstation (Siemens Medical, Germany), yielding mean transit time (MTT), cerebral blood volume (CBV), cerebral blood flow (CBF), and time to peak (TTP) maps. In addition, the CT stroke protocol was performed on a dual source CT scanner (Siemens Definition Flash). Pre- and post-contrast CT scans of the head were performed with the following parameters: 120 kilovolts (peak) (kVp), 220 mA (auto), 64_0.6 mm collimation, 0.28 s/rotation, and table speed of 1 mm/rotation. CT angiography was performed from the aortic arch to the vertex with the following parameters: total 60 cc of iodinated contrast agent was injected at 5 mL/s (Iohexol, Omnipaque, 350 mg iodine/mL; GE Healthcare, Piscataway, NJ), 5- to 10-seconddelay, 100/140kVp, auto mA, 0.28 s/rotation, 0.6-mm-thicksections, and table speed of 4 cm/rotation. CTA data were automatically processed by the technicians, including multiplanar 5 mm maximum intensity projection reconstructions and 5-mm axial reformates or CTA source images. The CTP technique included 45-second scanning reconstructed at 0.5-second intervals to produce a series of 90 sequential images for each of the 8 sections, covering a total of 40mm from the basal ganglia to the lateral ventricles. CTP scanning parameters were the following: 80 kVp, 150 mA, total 50 cc iodinated contrast agent injected at the rate of 5 mL/s.

### 2.7. Outcome assessment post bridging therapy

Primary outcome in this study was to identify any significant change in the functionality of patients who underwent treatment, which is done by assessment of NIHSS score, mRS score, and Barthel index both upon admission to the hospital and at 1-year (12 months) follow- ups. The regular followed at the outpatient settings were also documented 3, 6, 12 months post treatment. Additionally, using statistical methods, described in the following section, to determine whether any of the pre-intervention variables have a significant impact on the functional outcome.

### 2.8. Statistical analysis

Data was collected from 92 stroke patients with a total of 68 independent pre-interventional indexes and 4 outcome variables (Please refer to Tables S1 and S2, Supplemental Digital Content, http://links.lww.com/MD/H383) were recorded. Authors use principal component analysis (PCA) to integrate the prognostic variables into an aggregative index, prognosis, which is an index that takes into account all 4 outcome variables and their contribution to the overall outcome. Then using the forward stepwise regression model to determine which, if any, of the 68 independent variables had a significant effect on the prognosis. Authors end up with only eleven variables and only 7 of which had statistically significance.

#### 2.8.1 Classify the variables.

To facilitate the analysis, the data is first divided into pre-interventional and outcome variable, which are classified into categories, orders, and continuations. The outcome variables include the following: Follow up CT cerebral bleeding, outpatient recorded NIHSS, outpatient recorded mRS, outpatient recorded Barthel Index, and the remaining 68 variables are classified as pre-interventional independent variables (Tables S1 and S2, Supplemental Digital Content, http://links.lww.com/MD/H383).

#### 2.8.2 Utilization principal component analysis on the prognostic variables.

The authors use principal component analysis (PCA) to integrate the outcome variables into an aggregative index, prognosis. According to equation (1, see below), each principal component recombines the original variables into a new set of several independent variables. The coefficient of X_i_ in the linear combination is its eigenvector which is obtained by maximizing the explanatory variation of the corresponding principal component. Therefore, authors can determine the relationship between the principal component and the original variable.


$$\begin{array}{l} PCA=\phi_{1}X_{1}+\phi_{2}X_{2}+...+\phi_{n}X_{n} \end{array}$$ (1)

In the current study, prognosis is an outcome variable that is influenced by the need of follow up cerebral CT in case of suspected intracranial bleed, the NIHSS score, mRS score, and Barthel index. The relationship is shown in the below equation (2):


$$\begin{array}{l} Prognosis=0.218follow-upCT+0.566NIHSS+0.528mRS-0.595B.I \end{array}$$ (2)

#### 2.8.3 Exploratory data analysis.

The exploratory data analysis section was performed with the ggplot2 package in R. Authors describe the statistics of variables in order to understand the simple information of the data.

#### 2.8.4 Model architecture.

1. Multiple regression model

Multiple regression is to explore the correlation between the independent variable and the dependent variable and to build a regression model.^[6]^ The equation (3) was shown below.


$$\begin{array}{l} y=b_{0}+b_{1}x_{1}+b_{2}x_{2}+...+b_{n}x_{n}\left(b_{0}...b_{n}:Regressioncoef\;f\;icients\right) \end{array}$$ (3)

In our research, authors regarded all variables in pre-intervention as independent variables, and prognosis as a dependent variable, and built a multiple regression model. Authors find that only a few variables are significant, however, with Adj-*R*^2^ being only 0.3106, the model has a relatively low explanatory power. In order to improve the model, forward stepwise regression model is then used.

2. Forward stepwise regression model

The forward stepwise regression begins with an empty regression, adding variables one by one to select the best performing model according to the Akaike information criterion (AIC).^[7,8]^ AIC is a standard for assessing the complexity of statistical models and measuring the superiority of statistical model fit the data. The model with the lowest AIC value should be given priority when selecting the model. This is done by adding the independent variables one by one until the additional contribution of any one of the variables does not provide any statistical significance. Finally, the authors identified eleven variables with only 7 of them are significant (Table 3). This method improved the Adj-*R*^2^ of our model from 0.3106 to 0.4588, with *P* value <.05, and therefore, the model setup by using forward stepwise regression provides more explanatory power.

3. Segmented linear regression

Segmented linear regression is a method when the independent variables clustered into different groups.^[7]^ That is, there are different relationships between the variables in these regions. The following equation (4) is a threshold equation model:


$$\begin{array}{l} \eta =\alpha_{1}+{\alpha_{2}}^{T}z+\beta_{1}{\left(x-e\right)}_{+}+yx \end{array}$$ (4)

In this equation “e” is the threshold parameter, and “x” is the predictor with threshold effect, “z” denotes additional predictors.

Authors estimate a segmented linear regression model to determine if there is a threshold for independent variable age that would mark as a boundary for poor prognosis with statistical significance. The package authors utilized is chngpt package in R and uses the formula built in forward stepwise regression.

## 3. Study results

### 3.1. Exploratory data analysis

Table 1 shows the significantly pre-interventional predictive variables in acute ischemic stroke patients who underwent intravenous thrombolysis followed by intra-arterial thrombectomy.

**Table 1:** Pre-interventional independent important variables with statistical significance and distribution in a glance.

Admission Barthel Index	Age	Ipsilateral ICA RI	Ipsilateral VA RI
Min.: 0.00 1st Qu.: 5.00 Median:10.00 Mean:13.42 3rd Qu.:20.00 Max.:80.00	Min.:25.00 1st Qu.:55.75 Median:68.00 Mean:65.01 3rd Qu.:77.00 Max.:88.00	Min. :0.5100 1st Qu.:0.6400 Median:0.7000 Mean:0.7259 3rd Qu.:0.7625 Max.:1.7700	Min. :0.5300 1st Qu.:0.7000 Median:0.7850 Mean:0.7815 3rd Qu.:0.8500 Max.:1.0000
Contralateral MCA stenosis CTA	Contralateral ECA RI	In hospital pneumonia	
Min.:0.0000 1st Qu.:0.0000 Median:0.0000 Mean:0.3261 3rd Qu.:1.0000 Max.:1.0000	Min.:0.7400 1st Qu.:0.8175 Median:0.9100 Mean:0.9466 3rd Qu.:1.0000 Max.:2.3100	Min.:5.000 1st Qu.:8.000 Median: 9.000 Mean:8.413 3rd Qu.:9.000 Max.:10.000	

*Note*: These variables were identified using the forward stepwise regression model. Indicating that these 7 out of the total of 68 pre-interventional variables showed significant influence on the overall prognosis. Additionally, showing the minimum, maximum, median and quartile values of each variables.

CTA = computed tomography angiography, ECA = external carotid artery, ICA = internal carotid artery, MCA = middle cerebral artery, RI = resistance index, VA = vertebral artery.

The patients’ Barthel Index on admission is averaged to a value of 13.42 (range 0–80, lower the score means higher disability). This suggested that patients who presented with acute ischemic stroke were already disabled upon admission. The patients’ average age is 65, the youngest patient is 25, and the oldest patient is 88. It showed the majority of patients who suffered from stroke are elderly patients. The average value of ipsilateral internal carotid artery resistance index (ICA RI) is 0.7259, with a minimum value of 0.5100, and a maximum value of 1.7700. The average value of ipsilateral vertebral artery (VA) RI is 0.7815, with a minimum value of 0.5300, and a maximum value of 1.0000. The average value of contralateral external carotid artery (ECA) RI is 0.9466, with a minimum value of 0.7400, and a maximum value of 2.3100. This indicates that stroke was more likely to occur in a person with high arterial stagnation. The average value of contralateral middle cerebral artery (MCA) stenosis CTA is 0.3216, with a minimum value of 0.0000, and a maximum value of 1.0000. The average value of in-hospital pneumonia is 8.413, with a minimum value of 5.0000, and a maximum value of 10.0000.

Furthermore, admission Barthel Index, age, ipsilateral ICA RI, ipsilateral VA PI, contralateral ECA RI, contralateral MCA stenosis on computed tomography angiography (CTA), and in-hospital pneumonia are the most significant variables based on the dataset analysis (Table 1).

### 3.2. Multiple linear regression and forward stepwise regression

Table 2 showed the difference between the original model and forward stepwise regression model. The Adj-*R*^2^ of original model is 0.3106 and the *P* value of original model is .07819. The Adj-*R*^2^ of forward stepwise model is 0.4588 and the *P* value of forward stepwise model is 3.137e−09. The result showed that the forward stepwise model is superior to the original model.

**Table 2:** Model architecture of the data analysis in stratifying the baseline patients’ characteristics.

	Original model	Forward stepwise regression
Formula	Prognosis ~ all variables	Prognosis ~ Admission Barthel Index + Age + Ipsilateral ICA RI + Ipsilateral VA PI + Contralateral MCA stenosis CTA + Contralateral ECA RI + Stroke right/ left + In hospital pneumonia + MRA ipsilateral collateral flow + CTP mismatch + Admission CT ASPECTS
Residual standard error	1.189	1.054
Multiple *R*-squared	0.7954	0.5243
Adjusted *R*-squared	0.3106	0.4588
F-statistic	1.641	8.015
*P* value	.07819	3.137e−09 ***

*Note:* This table showed the difference between the original model using multiple regression and forward stepwise regression model. The Adj-*R*^2^ of original model is 0.3106 and the *P* value of original model was .07819. The Adj-*R*^2^ of forward stepwise model was 0.4588 and the *P* value of forward stepwise model was 3.137e−09.

ASPECTS = The Alberta stroke programme early CT score, CTA = computed tomography angiography, ECA= external carotid artery, ICA = internal carotid artery, MCA = middle cerebral artery, MRI = magnetic resonance imaging, PI = pulsatility index, RI = resistance index, VA, Vertebral artery.

In Table 3, it showed that admission Barthel Index (*P* value = .00212), age (*P* value = .03030), ipsilateral ICA RI (*P* value = .00155), ipsilateral VA PI (*P* value = .01274), contralateral MCA stenosis CTA (*P* value = .03008), contralateral ECA RI (*P* value = .02184) and in-hospital pneumonia (*P* value = .02126) are the significant variables.

**Table 3:** Pre-interventional variables in the forward stepwise regression analyses as to determining the important predictive power.

Categories	Coefficients	*P* value
Admission Barthel Index	−0.026867	.00212 **
Age	0.017807	.03030 *
Ipsilateral ICA RI	2.493430	.00155 **
Ipsilateral VA PI	0.214155	.01274 *
Contralateral MCA stenosis CTA	0.546799	.03008 *
Contralateral ECA RI	−1.148134	.02184 *
Stroke right/ left	−0.437726	.06663.
In-hospital pneumonia	0.561732	.02126 *
MRA ipsilateral Collateral flow	−0.449998	.06348.
CTP mismatch	0.006712	.11008
Admission CT ASPECTS	0.140513	.14005

Significant value: ***P value <.001; **P value <.01; *P value <.05; *P* value <.01. This table showed total of 11 variables identified using forward stepwise regression model, and 7 of the variables showed statistical significance. Admission Barthel Index (*P* value = .00212), Age (*P* value = .03030), Ipsilateral ICA RI (*P* value = .00155), Ipsilateral VA PI (*P* value = .01274), Contralateral MCA stenosis CTA (*P* value = .03008), Contralateral ECA RI (*P* value = .02184) and in-hospital pneumonia (*P* value = .02126) were the most significant variables.

ASPECTS = The Alberta stroke programme early CT score, CTA = computed tomography angiography, ECA= external carotid artery, ICA = internal carotid artery, MCA = middle cerebral artery, MRA = magnetic resonance arteriography, MRI = magnetic resonance imaging, PI = pulsatility index, RI = resistance index, VA, Vertebral artery.

Using the aforementioned equations (2) and (3), shown once again below. If the age is increased by 1 year, the prognosis value will increase by 0.017807, from equation (3). Then setting equation (2) is equal to equation (3), authors inferred that patients with older age were more likely to receive a followed- up CT of the head due to potential intracranial bleeding, a higher NIHSS and mRS scores, and lower Barthel Index score upon discharge. Using the same logic hypothesis, the authors arrive at the same conclusion with other variables such as ipsilateral ICA RI, Ipsi VA PI, contralateral MCA stenosis CTA, and in-hospital pneumonia.

Prognosis = 0.218(Followed-up CT) + 0.566(NIHSS) + 0.528(mRS) - 0.595(B.I)^[2]^

Prognosis = −0.026867(admission Barthel Index) + 0.017807(Age) + 2.493430(Ipsi ICA RI) + 0.214155(Ipsi VA PI) + 0.546799(Contralateral MCA stenosis CTA) - 1.148134(Contra ECA RI) - 0.437726(Stroke right left) + 0.561732(In-hospital pneumonia) - 0.449998 (magnetic resonance arteriography ipsilateral Collateral flow) + 0.006712(CTP mismatch) + 0.140513(Admission CT The Alberta stroke programme early CT score)^[3]^

On the contrary, if the value of contralateral ECA RI rises by value of one, the prognosis will decrease by 1.148134. That hints as below, the higher the value of contralateral ECA RI, the less chance having follow- up CT cerebral bleeding, and the better opportunity to full functional recovery. Other variables, admission Barthel index and magnetic resonance arteriography ipsilateral collateral flows values, show similar trends as above.

### 3.3. Segmented linear regression

**Figure 2.**
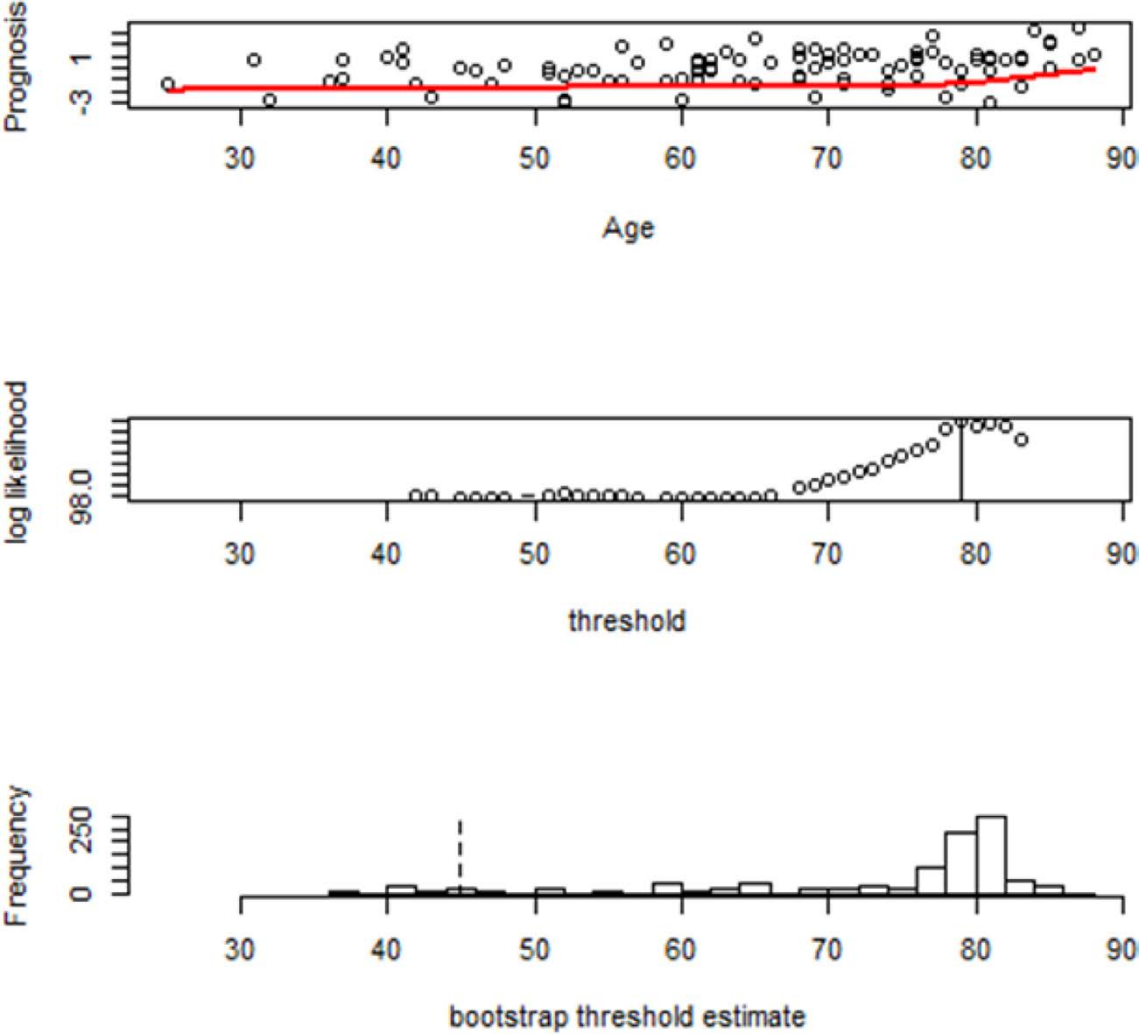
Threshold of the age among patients undergoing the intra-arterial thrombectomy treatment. The figure elucidates the 3 plots of the threshold of this data. The first plot shows the scatter plot between Age and Prognosis, and it represents that the samples scattered around 60 to 80 years old. The second plot shows the threshold of the Age variables is 79. The third plot shows the threshold which is estimated by bootstrap in order to know the frequency. We can presume the threshold for the variable “age” is 79.

Figure 2 showed the 3 plots of the threshold of this data. The first plot shows the scatter plot between Age and Prognosis, and it represents that the samples scattered around 60 to 80 years old. The second plot shows the threshold of the Age variables is 79. The third plot shows the threshold which is estimated by bootstrap in order to know the frequency. As we find that the frequency around 80 is the highest, thus we presume the threshold for the variable “age” set as 79.

According to the result of the segmented linear regression, the authors made the age beyond 79 as 1, and below 79 as 0, and conduct a simple linear regression to determine if there is a statistical difference between the 2 samples. The result is shown in Table 4, and it confirmed our presumption abovementioned that patients with age greater than 79 indeed having a worse prognosis than those who are younger than the age of 79 (*P* value = .008757).

**Table 4:** The linear regression for age threshold in differentiating the patients undergoing the intra-arterial thrombectomy long term functional outcome assessment.

	Model
Formula	Prognosis ~ Age
Residual standard error	1.386
Multiple *R*-squared	0.0739
Adjusted *R*-squared	0.06361
*F*-statistic	7.182
*P* value	.008757

Note: To verify the presumption derived from the scatter plot that age of 79 might be a threshold for poor prognosis, the authors divided age variables into 2 groups; the first group with age greater than 79, and the second group with age below 79, conducted via a simple linear regression. The result was shown in this table and confirmed that there was a significant difference in the prognosis between these 2 age groups.

## 4. Discussion

The current study demonstrated the higher the age, the less likely a stroke patient might recover from receiving treatment with intravenous thrombolysis followed by intra-arterial thrombectomy. This finding is especially resourceful for first-line clinicians in selecting candidate patients whom to receive such treatment and as a predictive tool to weigh out the benefit and risk. We believe this is the first paper that pinpointed the age threshold as an important indicator of functional outcome in acute ischemic stroke patients underwent such treatment.

As far as pre-intervention carotid doppler parameters, the higher the ipsilateral vertebral artery resistance index, which indicates compromised blood flow starting at the inlet of posterior circulation, therefore in the event of acute ischemic stroke of the anterior circulation, there are less available blood flow to rescue the ischemic brain cells and leading to higher risk of functional disability in such selected patients.^[9,10]^ As most of the collected patients more or less had the tandem stenosis both/or intra-cranially, the presence of the patent of the posterior vasculature stands as an important compensatory collateral flows that can minimize the stroke severity or post bridging therapy induced cerebral hemorrhagic or ischemic events

Interestingly, the current study showed that the higher the value of contralateral external carotid artery resistance index leads to a better functional outcome in ischemic stroke patients. We hypothesize that impaired contralateral blood flow re-directs blood flow toward the opposite side, which is the ischemic hemisphere, that is sufficient to prevent the progression of the disease and is meaningful in predicting the long-term functional outcome in patients.

The authors used different statistical analysis methods to identify specific variables and whether each had a significant impact on the overall prognosis. However, the Adj-*R*^2^ of the forward stepwise regression is only 0.4588, and the Adj-*R*^2^ of the simple linear regression is only 0.06361, which both can be attributed to the relatively small sample size (N = 92), therefore, the model had low explanatory power. It would increase our confidence in our observation if future studies with larger sample size and still showed similar trends.

The statistical analysis method we used was all supposed that there were linear relation between outcome variables and pre-interventional variables, therefore, authors use multiple linear regression and segmented regression as study models. However, it might be nonlinear relationship between 2 variables, and this should be considered in future studies to tailor consistency between the model and the real-world situation.

Based on the current literature, the golden stroke treatment strategy remains the intravenous thrombolytic bridging with intra-arterial thrombectomy. However, no all of the medical facility, in particular the rural areas around the world, could be under great medical insufficiency due to either lack of medical man power or surgery technical training. The cut off point for this group of stroke patient are still treated with empirical experiences and their long-term outcome is yet to be thoroughly investigated. Though 65-year-old, in general has been recognized a cutoff point age that could differentiate between younger or elder age group, the treatment long term outcome could not be applied successfully on the stroke patients receiving bridging therapy. There exists a gap between real world reality and textbook statics information.^[11]^

A major strength of this study is low heterogeneity among participants, given that most patients came from the surrounding local communities and shared the same ethnic group. Other strengths were technical consistency, as the same technician performed all of the carotid duplex scans, and the ability to compare each patient’s functional scores 1 year after the treatment allowed adequate time to also observe functional improvement. However, this could also potentially introduce confounding factors, such as different types of rehabilitation programs that participants may have attended within the year. The major shortcoming of the current study is of relatively small sample size (N = 92) as well as the fact there was no placebo group for comparison. Besides, the homogeneity in the ethnic group might not be able to represent other ethnic groups and therefore the general application can be limited in such regard.

Our current study shows several highlights listed as bellows, the higher the age, in-hospital pneumonia, contralateral middle cerebral artery stenosis on computed tomographic arteriography, ipsilateral internal carotid artery resistance index and ipsilateral vertebral artery pulsatility index, are correlated with higher risk of functional disability, whereas higher the value of contralateral external; carotid artery resistance index and Barthel Index on admission showed relative better likelihood in improving functional outcome in the long-term in acute ischemic stroke patients who underwent intravenous thrombolysis followed by intra-arterial thrombectomy (bridging therapy).

Pre-intervention parameters can potentially serve as important indicators to aid first-line clinicians in decision making. Especially, in patients whose age is above 79 receive diminished return in the benefit to undergo such intervention and should be considered seriously by both the patients and the physicians.

## Acknowledgment

This work was supported in part by the National Science and Technology Council, Taiwan, R.O.C., under Grant no. MOST 110-2118-M-A49-002-MY3 and MOST-110-2634-F-A49-005, in part by the Higher Education Sprout Project of the National Yang Ming Chiao Tung University and Ministry of Education, Taiwan, R.O.C., and in part by Ministry of Education Yushan Scholar Program, Taiwan, R.O.C. We are grateful to the National Center for High-performance Computing for computer time and facilities. Our team would like to thank Ms Giulia Mengato for her proofreading.

The authors have no conflicts of interest to disclose.

## Author contributions

Writing – review & editing: Yang-Hao Ou and Chih Ming Lin
